# Overexpression of caudal-related homeobox transcription factor 2 inhibits the growth of transplanted colorectal tumors in nude mice

**DOI:** 10.3892/mmr.2015.3838

**Published:** 2015-05-25

**Authors:** JIAN-BAO ZHENG, LI-NA QIAO, XUE-JUN SUN, JIE QI, HAI-LIANG REN, GUANG-BING WEI, PEI-HUA ZHOU, JIAN-FENG YAO, LI ZHANG, PENG-BO JIA

**Affiliations:** 1 Department of General Surgery, First Affiliated Hospital of Medical College, Xi'an Jiaotong University, Xi'an, Shaanxi 710061, P.R. China; 2 Second Department of Cardiovascular Medicine, Shaanxi Provincial People's Hospital, Xi'an, Shaanxi 710068, P.R. China; 3 Department of General Surgery, The Third Hospital of Chengdu, Chengdu, Sichuan 610031, P.R. China

**Keywords:** matrix metalloproteinase-2, caudal-related homeobox protein, subcutaneously-transplanted tumor, colorectal cancer

## Abstract

Caudal-related homeobox transcription factor 2 (CDX2) is a transcription factor, which is specifically expressed in the adult intestine. It is essential for the development and homeostasis of the intestinal epithelium and its functions as a tumor suppressor have been demonstrated in the adult colon. The present study aimed to examine the inhibitory effects of the overexpression of CDX2 on subcutaneously-transplanted tumors, derived from LoVo colon cancer cells, in nude mice, and to provide experimental evidence for the biotherapy of colon cancer. A pEGFP-C1-CDX2 eukaryotic expression vector was transfected into the LoVo cells via lipofection, and LoVo cells stably-expressing CDX2 (pEGFP-C1-CDX2 cells) were obtained using G418 selection. A nude mouse subcutaneously-transplanted tumor model was established by inoculating the nude mice with the pEGFP-C1-CDX2 cells, and the effects of overexpression of CDX2 on transplanted tumor growth in the LoVo cells were observed. Western blotting results demonstrated that the protein expression of CDX2 in the LoVo cells was higher in the pEGFP-C1-CDX2 cell group, compared with that in the pEGFP-C1 cell group and the untreated cell group. At 20 days post-inoculation with either pEGFP-C1-CDX2 or pEGFP-C1, the transplanted tumor masses were significantly lower in the pEGFP-C1-CDX2 group, compared with those in the pEGFP-C1 and untreated groups. Immunohistochemistry revealed that the expression levels of CDX2 and matrix metalloproteinase-2 (MMP-2) were detected in each group, and the protein expression of CDX2 was increased in the tumor tissues from the nude mice in the pEGFP-C1-CDX2 group. However the expression of MMP-2 was downregulated in the tumor tissues of the nude mice in the pEGFP-C1-CDX2 group. Taken together, these data suggested that pEGFP-C1-CDX2 cells exhibited suppressed tumor growth *in vivo*. Overexpression of CDX2 was observed in transplanted tumors in the pEGFP-C1-CDX2 group, and the gene expression of MMP-2 was reduced. These results indicate that CDX2 inhibited the growth of colorectal tumor cells, possibly by downregulating the gene expression.

## Introduction

Colorectal cancer is the third most common type of cancer and is the third leading cause of cancer-associated mortality in males and females in the United States (1). However, despite significant effort, the molecular pathways involved, and the order of genetic events in the genesis of colorectal cancer remain to be fully elucidated. Caudal-related homeobox transcription factor 2 (CDX2) is a transcription factor, which is specifically expressed in the adult intestine. It is essential for the development and homeostasis of the intestinal epithelium (2). CDX2 is central in the regulation of the balance between differentiation and proliferation of intestinal epithelial cells (IECs) (3). Conditional intestine-specific inactivation of the murine CDX2 gene has a marked effect on the villus morphology and cytodifferentiation of IECs (4). Previous chromatin immunoprecipitation-sequencing data has revealed that CDX2 binds a significantly higher number of target genes in differentiated IECs, compared with proliferating cells (5,6). In adult human tissue, a number of studies have identified the involvement of CDX2 in regulating the expression of genes encoding intestine-specific proteins, including sucrase-isomaltase (7), lactase (8,9), calbindin-D9K (10,11), apolipoprotein B (12), claudin-2 (13) and mucin 2 (14,15). Additionally, it has been demonstrated that CDX2 functions as a tumor suppressor in the adult colon. Bonhomme *et al* revealed that reduced expression of CDX2 accelerates tumor progression in a mouse model of sporadic colorectal cancer (16). Furthermore, Aoki *et al* confirmed these findings in a mouse model of familial adenomatous polyposis (17). The role of CDX2 as a tumor suppressor is also supported by the observation that its expression is decreased in human colorectal cancer, and reduced expression of CDX2 is associated with poor overall survival rates in patients with colorectal cancer (18–20). Histopathological analyses have demonstrated that the expression of CDX2 is low in invasive colorectal cancer cells, which localize at the tumor/stroma interface, but is restored in metastases, at a level corresponding to that of the primary tumor (21). These data suggest that decreased expression of CDX2 is involved in tumor migration. In the present study, the effects of the overexpression of CDX2 on the growth of colon cancer was were investigated via subcutaneous implantation of CDX2-overexpressing LoVo colon cancer cells, delivered using a transfected eukaryotic expression vector, pEGFP-C1-CDX2, into a nude mouse model.

## Materials and methods

### Cell line and culture

The LoVo human colon cancer cell line was purchased from China Centre for Type Culture Collection (Shanghai, China). The cells were cultured at 37°C in RPMI-1640 medium (Gibco Life Technologies, Grand Island, NY, USA), supplemented with 10% fetal bovine serum (Hyclone, Waltham, MA, USA) in a humidified atmosphere of 5% CO_2_. The cells were detached using 0.25% trypsin and 0.02% ethylenediaminetetraacetic acid (EDTA) (Boster Biological Technology Co., Ltd., Wuhan, China).

### Animals

Athymic nude male BALBC/c mice, weighing 15–18 g (4–5 weeks old), were purchased from the Institute of Laboratory Animal science, Chinese Academy of Medical Science (Beijing, China). The mice were maintained in specific pathogen-free, temperature-controlled (24°C) conditions, were housed separately and were fed with sterilized food and autoclaved water, according to the experimental animal guidelines (22). All animal procedures were approved by the Committee on Animal Experimentation of Xi'an Jiaotong University (Xi'an, China), and the procedures complied with the NIH Guide for the Care and Use of Laboratory Animals (23).

### Vector construction and transfection

CDX2 full-length cDNA was amplified using reverse transcription-polymerase chain reaction (RT-PCR) using total RNA, which was extracted according to the manufacturer's instructions using TRIzol (Gibco Life Technologies) from human colorectal carcinoma LoVo cells as a template. The resulting RNAs were treated with RNase-free DNase (Promega Corporation, Madison, WI, USA) and 2.5 *µ*g was reverse transcribed into cDNA using the RT-PCR kit (Invitrogen Life Technologies, Carlsbad, CA, USA) according to the manufacturer's instructions. The following primers were used: Forward 5′-CCA ATA AGC TTA GGC AGC ATG GTG AGG TCT G-3′) and reverse 5′-CCA ATG GAT CCC TGA GGA GTC TAG CAG AGT CCA C-3′. PCR amplifications were performed in duplicate and the conditions were as follows: Initial denaturation at 94°C for 2 min, then 35 cycles of 94°C for 30 sec, 62°C for 30 sec and 72°C for 30 sec, followed by an extension step at 72°C for 10 min. Full-length CDX2 cDNA (1,055 bp) was then cloned into the *Hind*II/*Bam*HI sites of the pEGFP-C1 eukaryotic expression vector, (Clontech Laboratories, Inc., Mountain View, CA, USA). The LoVo cells were transfected using Lipofectamine™ 2000 (Invitrogen Life Technologies), according to the manufacturer's instructions. The cells (1×10^5^ cells/cm^2^ in 24-well plates) were then grown in complete medium containing 250 mg/ml G418 (Sigma-Aldrich, St. Louis, MO, USA) at 37°C. Subsequent to transient transfection for 48 h, the cells were passaged at 1:10 (volume/volume) and cultured in medium supplemented with G418 (Sigma-Aldrich) at 600 *µ*g/ml for 4 weeks. The survival clones were selected and maintained in medium containing 300 *µ*g/ml G418. The subclone cells expressing CDX2 were termed the pEGFP-C1-CDX2 cells. CDX2 cloning was confirmed using western blotting.

### Western blotting

For western blotting, ~1×10^7^ untreated cells, pEGFP-C1 cells and pEGFP-C1-CDX2 cells were harvested, washed once with ice-cold phosphate-buffered saline (PBS; Boster Biological Technology Co., Ltd.), resus-pended in 100–200 *µ*l lysis buffer (Sigma-Aldrich), containing 50 mM Tris, 150 mM NaCl, 5 mM EDTA, 5 mM ethylene glycol tetraacetic acid and 1% SDS (pH 7.5), and then ultrasonicated (MS2 Minishaker; IKA-Works, Wilmington, NC, USA) on ice until the solution became clear. The total protein concentration was measured using the Bradford method (24), according to the manufacturer's instructions (Sigma-Aldrich). The samples were heated at 100°C for 5 min with and equal volume of 2X SDS loading buffer (Shaanxi Pioneer Biotech Co., Ltd., Xi'an, China), containing 125 mM Tris-HCl (pH 6.8), 10% glycerol, 2% SDS, 5% 2-mercaptoethanol and 2.5 ml 0.0025% bromophenol blue, and were cooled on ice for 10~20 min. Total protein (80 *µ*g) from each sample was resolved using 8% or 10% SDS-PAGE to detect CDX2 (38 kDa) and β-actin (43 kDa), respectively. The protein was then transferred onto a polyvinylidine difluoride membrane in transfer buffer, containing 25 mM Tris (pH 8.5), 200 mM glycerin, and 20% methanol) at 100 V for 2 h. The proteins were detected using mouse monoclonal CDX2 antibody (cat. no. AM392-M; 1:500; BioGenex, San Ramon, CA, USA) and mouse monoclonal β-actinantibody (cat. no. sc-47778; 1:1,000, Santa Cruz Biotechnology, Inc., Santa Cruz, CA, USA) as a loading control. The membranes were incubated with primary antibodies overnight at 4°C after blocking in 5% non-fat milk for 1 h at room temperature, and were then incubated with horseradish peroxidase-conjugated goat anti-rabbit or goat anti-mouse immunoglobulin G (Beijing ZhongShan Goldbridge Biotechnology Co, Beijing, China). The proteins were visualized using chemiluminescence luminol reagents (cat. no. sc-2048; Santa Cruz. Biotechnology, Inc.).

### Subcutaneous human colorectal cancer cell xenograft growth and oncogenicity

A total of 18 nude mice were randomly divided into three groups (n=6), comprising an untreated cell group, a group inoculated with pEGFP-C1 cells and a group inoculated with pEGFP-C1-CDX2 cells. The mice were subcutaneously inoculated in the right hind lateral leg with cells (1×10^7^/ml) in the logarithmic growth period. At 10 days post-injection, tumor growth was monitored every 2 days. The measurements of the tumor diameter (a) and short diameter (b), measured using a vernier caliper (Qingdao Tide Machine Tool Supply Co., Ltd., Qingdao, China), were used to calculate the tumor volume, according to the formula a × b^2^ / 2, and tumor growth curves were drawn on the basis of the average values of the tumor volume from each group. At the end of the third week, the mice were sacrificed by cervical dislocation, and the tumors were resected in order to measure the final volumes and weights using a photoelectric balance (ACS-JL808 LED; Yongkang Jieli Weighing Apparatus Co., Ltd, Yongkang, China). The tumor growth inhibition rate was determined using the volume and weight measurements and the following formula: Inhibition rate = (1 - tumor weight of transfectant / tumor weight of untreated cells) × 100%. The tumor tissue of each group was stored in liquid nitrogen. A total of 18 sections (0.25 cm^3^) of the tissues were fixed with 10% formaldehyde (Boster Biological Engineering Co., Ltd.) solution for subsequent immunohistochemical analysis.

### Immunohistochemical staining

The tumor tissues were fixed in 4% formaldehyde, dehydrated using gradient ethanol, and embedded in paraffin (Xian Chemical Reagents Instruments, Inc., Xian, P.R. China). Tissue sections (4 *µ*m) were deparaffinized in fresh xylene (Xian Chemical Reagents Instruments, Inc.) and rehydrated through sequential graded ethanols. Antigen retrieval was performed by incubation with citrate buffer (10 mmol/l; pH 6.0) using a microwave pressure cooker (Zhejiang Duobao Industrial & Trade Co., Ltd., Ningbo, China) for 20 min. The slides were cooled for 20 min, incubated for 5 min with 3% hydrogen peroxide (Xian Chemical Reagents Instruments, Inc.), washed in PBS-0.1% Triton X-100 (pH 7.6), blocked for 20 min in 20% normal goat serum (Boster Biological Engineering Co., Ltd.), and incubated in an appropriate antibody dilution for CDX2 (cat. no. MU392A-UC; 1:400; mouse monoclonal; Biogenex, San Ramon, CA, USA) or MMP-2 (cat. no. BA0569; 1:400; rabbit polyclonal; Boster Biological Technology Co., Ltd.) overnight at 4°C. The subsequent day, the slides were washed in PBS-0.1% Triton X-100 and incubated for 30 min in a 1:200 dilution of biotinylated anti-mouse (cat. no. BA1001) or anti-rabbit (cat. no. BA1003) secondary antibody. The ABC Elite kit (Boster, Biological Technology Co., Ltd.), with 3,3′-diaminobenzidine development, was used to visualize antibody binding, and the slides were subsequently counterstained with hematoxylin (0.4%; Boster Biological Engineering Co., Ltd.). Negative controls were included by replacement of the primary antibody with PBS. Evaluation of the immunostaining of the CDX2 and MMP-2 genes were performed simultaneously by two independent observers in a blinded manner using an Olympus BX51 microscope (Olympus, Center Valley, PA, USA).

### Statistical analysis

All data are expressed as the mean ± standard error of the mean. Differences were assessed between the two groups using a t-test. P<0.05 was considered to indicate a statistically significant difference. All statistical analyses were performed using SPSS software, version 13.0 (SPSS Inc., Chicago, IL, USA).

## Results

### Construction of the pEGFP-C1-CDX2 eukaryotic vector and overexpression of CDX2 in LoVo cells

**Figure 1:**
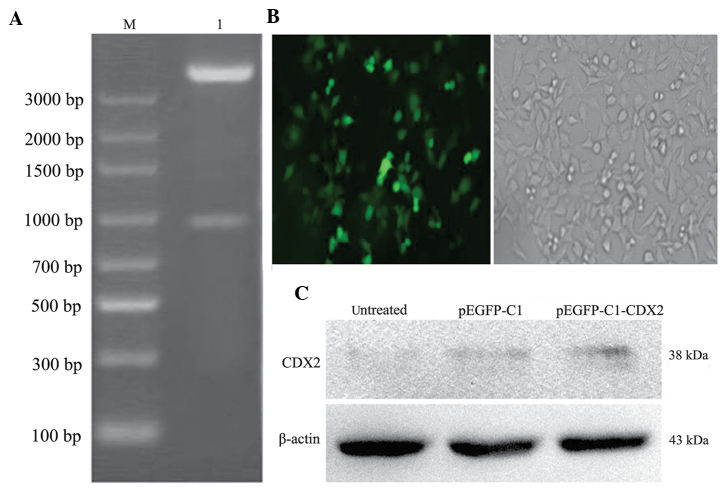
Identification of the pEGFP-C1-CDX2 eukaryotic expression vector and overexpression of CDX2 in the LoVo cells. (A) pEGFP-C1-CDX2 was identified using a double enzyme digestion assay with *Hind*II/*Bam*HI. The DNA fragments of 1,055 bp (CDX2) and 4.7 kb (pEGFP-C1) were amplified. (B) Monoclones of the pEGFP-C1-CDX2 cells were visualized using light microscopy (left) and fluorescence microscopy (right). Magnification, ×100. (C) Protein expression of CDX2 was detected using western blotting. The expression of β-actin was used as internal control. M, marker; 1, double enzyme digestion; CDX2, caudal-related homeobox transcription factor 2.

The pEGFP-C1-CDX2 recombinants were validated using DNA sequencing analysis (data not shown) and restriction endonuclease analysis (Fig. 1A). None of the untransfected LoVo cells survived following G418 (250 *µ*g/ml) selection for 2 weeks. The pEGFP-C1-CDX2- and pEGFP-C1-transfected cells were continuously selected using G418 for 6 weeks, until a mono-clone was observed (Fig. 1B). The clones were then amplified, to provide subclone pEGFP-C1 cells, pEGFP-C1-CDX2 cells and untreated cells.

To investigate the protein expression levels of CDX2 in the untreated cells, pEGFP-C1 cells and pEGFP-C1-CDX2 cells, the levels of CDX2 were measured using western blotting. The relative expression levels of CDX2 to β-actin were determined. The protein level of CDX2 in the pEGFP-C1-CDX2 cells was significantly higher than those in the untreated cells and pEGFP-C1 cells (P<0.05). The brightness of the CDX2 bands between the untreated and pEGFP-C1 cells exhibited no significant difference (P>0.05; Fig. 1C).

### Growth of xenograft tumors in nude mice

**Figure 2:**
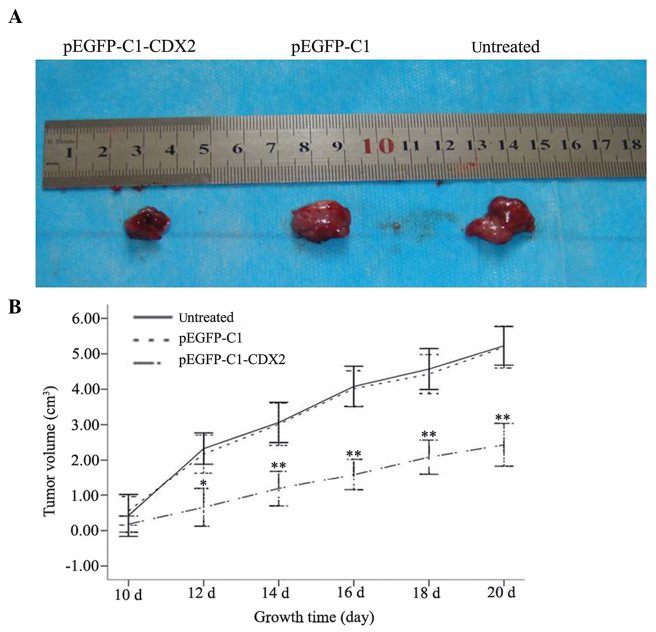
Overexpression of the CDX2 gene inhibits tumour growth of LoVo cells *in vivo*. (A) Representative images of the tumors isolated from three mice 3 weeks after inoculation with either untreated cells, pEGFP-C1 cells or pEGFP-C1-CDX2 cells (n=3/group). (B) Overexpression of CDX2 inhibited tumour growth of the LoVo cells *in vivo* (n=6/group). Data are expressed as the mean ± standard error of the mean. ^*^P<0.05 and ^**^P<0.01 vs. untreated and pEGFP-C1 cell groups. CDX2, caudal-related homeobox transcription factor 2.

Within an average of 10 days, a 3- to 4-mm diameter tumor developed at the subcu taneous injection sites of the right hind lateral leg of the nude mice, with a 100% tumor formation rate. From day 12, the tumor volumes between the pEGFP-C1-CDX2 cell group and the untreated cell and pEGFP-C1 cell groups were significantly different (P<0.05) and by day 20, tumor volumes of 5.22±0.27, 5.19±0.30 and 2.43±0.30 cm^3^ were recorded in the untreated, pEGFP-C1 and pEGFP-C1-CDX2 cell groups, respectively (Fig. 2A). Observations during the 10 day period were performed and a tumor growth curve was plotted (Fig. 2B). The nude mice in each group were healthy, fed a normal diet and exhibited no toxicity throughout the feeding process during the inhibition of LoVo proliferation by overexpressing CDX2. Until sacrifice of the nude mice on day 21, the average weights of the transplanted tumors in the untreated, pEGFP-C1 and pEGFP-C1-CDX2 groups were 0.62±0.22, 2.10±0.78 and 2.56±0.76 g, respectively. The tumor weight in the pEGFP C1 CDX2 group was significnatly lighter than the other two groups (^*^P<0.05). The rate of inhibition of tumor weight in the pEGFP-C1-CDX2 cells group was 75.79%, which was increased compared with the pEGFP-C1 group (17.79%) (Table I).

### Immunohistochemical analysis of the protein expression levels of CDX2 and MMP-2 in the xenograft tumors

**Figure 3:**
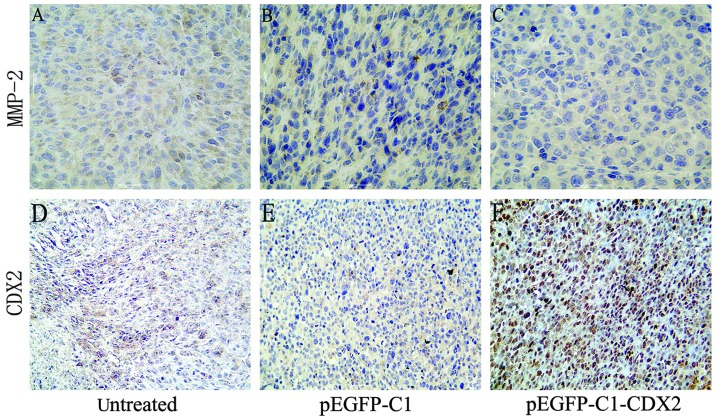
Immunohistochemical analysis of MMP-2 and CDX2 proteins in LoVo xenograft tumors. The sections were immunostained with 3,3′-diaminobenzidine and counterstained with hematoxylin for the expression of MMP-2 and CDX2. Representative samples are shown (A–C) magnification, ×400; (D–F) magnification, ×200. It was observed that MMP-2 was expressed in the cytoplasm in LoVo xenograft tumors, and that CDX2 was expressed in the nucleus. MMP2, matrix metalloproteinase-2; CDX2, caudal-related homeobox transcription factor 2.

The protein expression levels were also investigated using immunohistochemical staining of the xenograft tumor tissues. An increase in the expression of CDX2, which was distributed in the nucleus, was observed in the stably pEGFP-C1-CDX2-transfected cells (brown staining in Fig. 3). In addition, the immunohistochemical staining revealed a decrease in the protein expression of MMP-2 in the pEGFP-C1-CDX2 cell group, compared with the untreated and pEGFP-C1 cell groups.

## Discussion

CDX2 is a member of the caudal homeobox gene family. It was identified over 10 years ago in *Drosophila melanogaster*. CDX2 is essential in intestinal epithelial cell proliferation, differentiation, formation and maintenance of columnar morphology and polarization (25,26), and is an intestinal epithelial-specific marker with high sensitivity in the human body. Excluding the intestinal epithelium, the expression of CDX2 is seldom detected in the normal epithelium of other systems (27). CDX2 is a tumor suppressor gene in the formation and development of tumors, however, its antitumor mechanism remains to be fully elucidated. CDX2 may be involved in the arrest of cell differentiation, abnormal decreased proliferation and altered cell adhesion. Our previous study demonstrated that the positive rate of CDX2 was 69.4% in colorectal cancer, and 95.0% in normal colorectal tissues, suggesting that the expression of CDX2 was high in the colorectal tissues. In addition, the expression of CDX2 was significantly higher in the normal colorectal tissues, compared with the colorectal cancer tissues. With the decreased degree of colorectal cancer differentiation, the protein expression of CDX2 appeared reduced. The protein expression of CDX2 was also markedly lower in the lymph node metastasis group than in the non-lymph node metastasis group. In addition, CDX2 expression is reduced in Dukes A-D colorectal cancer (28). These results indicated that CDX2 possibly acts in the development of colorectal cancer. The expression of CDX2 is correlated with the degree of colorectal cancer malignancy, and decreased expression of CDX2 indicates an increased degree of tumor malignancy and increased invasion and metastasis, which has been observed in previous studies (19,29). The above-described results suggest that decreased expression of CDX2 is the predominant reason for differentiation arrest or loss of tumor cells, and increased expression levels of CDX2 possibly inhibit the growth and metastasis of tumor cells. However, the above-mentioned studies were focused predominantly on immunohistochemical expression and *in vitro* molecular biological effects, while few studies have investigated the *in vivo* effects.

The present study focused on *in vivo* experiments in nude mice. Stably-transfected pEGFP-C1-CDX2 cells, negative control pEGFP-C1 cells and untreated LoVo cells were injected into BALB/c nude mice. Subcutaneous tumor formation was detected within 10 days. With prolonged duration, tumor growth was slower in the pEGFP-C1-CDX2 group compared with the pEGFP-C1 and untreated control groups, with statistical significance. However, no significant difference in tumor growth was observed between the pEGFP-C1 and untreated cells groups. These results confirmed that the overexpression of CDX2 in LoVo cells repressed the growth of transplanted tumors.

The present study revealed that the overexpression of CDX2 inhibited the proliferation of LoVo tumor cells *in vivo*. However, the results of our previous *in vitro* investigation demonstrated that the inhibitory effect on the LoVo cells was not significantly altered following overexpression of CDX2, with no suppression of cell division, indicating that CDX2 may not be involved in the proliferation of colon cancer cells (30). The conflicting results between the present *in vivo* experiments and previous *in vitro* experiments may be due to a number of reasons During the *in vitro* experiments, CDX2 was transiently expressed in the LoVo cells. MTT assays were used to measure the effects of CDX2 on tumor cell proliferation within 72 h, as well as on the cell cycle and apoptosis. The *in vivo* experiments were performed over a loner time-period, indicating that the inhibitory effects of CDX2 on colorectal cancer were time-dependent and the short-term effects were not significant, but CDX2 may inhibit tumor growth over a longer duration. In addition, *in vivo* experiments are more representative of the microenvironment of human body, which is composed of tumor cells, various host cells, extracellular matrix and abundant secreted factors. The extracellular matrix affects tumor cell proliferation, invasion and metastasis by affecting the gene expression of CDX2 (31–33). Additionally, hypoxic microenvironments commonly occur in solid tumors, and hypoxia impacts the growth of these tumors by affecting the gene expression of CDX2 (28,34). However, the conclusion of the present study was consistent with our previous conclusion that low gene expression levels of CDX2 in colorectal cancer accelerates the malignant growth of colorectal tumors (28).

Invasion and metastasis are basic biological characteristics of malignant tumor cells, and >80% patients with cancer succumb to mortality from these. Tumor cells have to degrade the extracellular matrix and basement membrane during local invasion and distant metastasis. Degradation of the extracellular matrix depends on predominantly on various proteolytic enzymes (35). Of the MMPs, MMP-2 is important in tumor invasion and metastasis due to the ability of MMP-2 to specifically degrade collagen IV (36). A previous study verified that MMP-2 affects the immune response and extracellular matrix remodeling, promotes formation of tumor angiogenesis and contributes to tumor metastasis (37). Numerous studies have confirmed that increased expression of MMP-2 is positively correlated with the invasion and metastasis of human gastric cancer, colon cancer and breast cancer (38–43). In the present study, protein expression of CDX2 was observed in the tumor tissues following inoculation with the pEGFP-C1-CDX2 cells. In addition, the protein expression of MMP-2 was significantly reduced, suggesting that CDX2 suppressed the invasion and metastasis of tumor cells, possibly by downregulating the expression of MMP-2, which was also demonstrated in the results of our previous *in vitro* investigation (31).

In conclusion, CDX2 exhibited notable inhibitory effects on the progression of colorectal cancer. CDX2 may be an important tumor suppressor, which can be used as a novel index to screen and monitor colorectal cancer, and may provide a novel strategy for targeted cancer therapy.

## Figures and Tables

**Table I:** Tumor parameters following the 10-day period prior to sacrifice of the nude mice.

Cell group	Tumor weight (g)	Inhibition rate (%)
pEGFP-C1-CDX2	0.62±0.22	75.79
pEGFP-C1	2.10±0.78*	17.79
Untreated	2.56±0.76*	–

Data are expressed as the mean ± standard error of the mean.

* P<0.05; pEGFP-C1-CDX2 vs. pEGFP-C1 cells, pEGFP-C1-CDX2 vs. untreated cells. Inhibition rate = (1 - tumor weight of transfectant / tumor weight of untreated cells) ×100%. CDX2, caudal-related homeobox transcription factor 2.

## Abbreviations

CDX2: caudal-related homeobox protein 2
IECs: intestinal epithelial cells
MMP-2: matrix metalloproteinase-2
CRC: colorectal cancer

## References

[1] Siegel R, Ma J, Zou Z, Jemal A (2014). Cancer statistics, 2014. CA Cancer J Clin.

[2] Beck F, Chawengsaksophak K, Waring P, Playford RJ, Furness JB (1999). Reprogramming of intestinal differentiation and intercalary regeneration in Cdx2 mutant mice. Proc Natl Acad Sci USA.

[3] Olsen AK, Boyd M, Danielsen ET, Troelsen JT (2012). Current and emerging approaches to define intestinal epithelium-specific transcriptional networks. Am J Physiol Gastrointest Liver Physiol.

[4] Gao N, White P, Kaestner KH (2009). Establishment of intestinal identity and epithelial-mesenchymal signaling by Cdx2. Dev Cell.

[5] Verzi MP, Shin H, He HH, Sulahian R, Meyer CA, Montgomery RK, Fleet JC, Brown M, Liu XS, Shivdasani RA (2010). Differentiation-specific histone modifications reveal dynamic chromatin interactions and partners for the intestinal transcription factor CDX2. Dev Cell.

[6] Boyd M, Hansen M, Jensen TG, Perearnau A, Olsen AK, Bram LL, Bak M, Tommerup N, Olsen J, Troelsen JT (2010). Genome-wide analysis of CDX2 binding in intestinal epithelial cells (Caco-2). J Biol Chem.

[7] Suh E, Chen L, Taylor J, Traber PG (1994). A homeodomain protein related to caudal regulates intestine-specific gene transcription. Mol Cell Biol.

[8] Troelsen JT, Mitchelmore C, Spodsberg N, Jensen AM, Norén O, Sjöström H (1997). Regulation of lactase-phlorizin hydrolase gene expression by the caudal-related homoeodomain protein Cdx-2. Biochem J.

[9] Fang R, Santiago NA, Olds LC, Sibley E (2000). The homeodomain protein Cdx2 regulates lactase gene promoter activity during enterocyte differentiation. Gastroenterology.

[10] Lambert M, Colnot S, Suh E, L'Horset F, Blin C, Calliot ME, Raymondjean M, Thomasset M, Traber PG, Perret C (1996). cis-Acting elements and transcription factors involved in the intestinal specific expression of the rat calbindin-D9K gene: binding of the intestine-specific transcription factor Cdx-2 to the TATA box. Eur J Biochem.

[11] Colnot S, Romagnolo B, Lambert M, Cluzeaud F, Porteu A, Vandewalle A, Thomasset M, Kahn A, Perret C (1998). Intestinal expression of the calbindin-D9K gene in transgenic mice. Requirement for a Cdx2-binding site in a distal activator region. J Biol Chem.

[12] Lee SY, Nagy BP, Brooks AR, Wang DM, Paulweber B, Levy-Wilson B (1996). Members of the caudal family of home-odomain proteins repress transcription from the human apolipoprotein B promoter in intestinal cells. J Biol Chem.

[13] Sakaguchi T, Gu X, Golden HM, Suh E, Rhoads DB, Reinecker HC (2002). Cloning of the human claudin-2 5′-flanking region revealed a TATA-less promoter with conserved binding sites in mouse and human for caudal-related homeodomain proteins and hepatocyte nuclear factor-1alpha. J Biol Chem.

[14] Yamamoto H, Bai YQ, Yuasa Y (2003). Homeodomain protein CDX2 regulates goblet-specific MUC2 gene expression. Biochem Biophys Res Commun.

[15] Mesquita P, Jonckheere N, Almeida R, Ducourouble MP, Serpa J, Silva E, Pigny P, Silva FS, Reis C, Silberg D, Van Seuningen I, David L (2003). Human MUC2 mucin gene is transcriptionally regulated by Cdx homeodomain proteins in gastrointestinal carcinoma cell lines. J Biol Chem.

[16] Bonhomme C, Duluc I, Martin E, Chawengsaksophak K, Chenard MP, Kedinger M, Beck F, Domon-Dell C (2003). The Cdx2 homeobox gene has a tumour suppressor function in the distal colon in addition to a homeotic role during gut development. Gut.

[17] Aoki K, Tamai Y, Horiike S, Oshima M, Taketo MM (2003). Colonic polyposis caused by mTOR-mediated chromosomal instability in Apc+/Delta716 Cdx2+/-compound mutant mice. Nat Genet.

[18] Bakaris S, Cetinkaya A, Ezberci F, Ekerbicer H (2008). Expression of homeodomain protein CDX2 in colorectal adenoma and adenocarcinoma. Histol Histopathol.

[19] Choi BJ, Kim CJ, Cho YG, Song JH, Kim SY, Nam SW, Lee SH, Yoo NJ, Lee JY, Park WS (2006). Altered expression of CDX2 in colorectal cancers. APMIS.

[20] Hong KD, Lee D, Lee Y, Lee SI, Moon HY (2013). Reduced CDX2 expression predicts poor overall survival in patients with colorectal cancer. Am Surg.

[21] Brabletz T, Spaderna S, Kolb J, Hlubek F, Faller G, Bruns CJ, Jung A, Nentwich J, Duluc I, Domon-Dell C, Kirchner T, Freund JN (2004). Down-regulation of the homeodomain factor cdx2 in colorectal cancer by collagen type I: an active role for the tumor environment in malignant tumor progression. Cancer Res.

[22] American Psychological Association Committee on Animal Research Ethics (2012). Guidelines for ethical conduct in the care and use of nonhuman animals in research.

[23] National Research Council (US) Committee for the Update of the Guide for the Care and Use of Laboratory Animals (2011). Guide for the Care and Use of Laboratory Animals.

[24] Hammond JB, Kruger NJ (1988). The bradford method for protein quantitation. Methods Mol Biol.

[25] Kawai H, Tomii K, Toyooka S, Yano M, Murakami M, Tsukuda K, Shimizu N (2005). Promoter methylamine down regulates CDX2 expression in colorectal carcinomas. Oncol Rep.

[26] Keller MS, Ezaki T, Guo RJ, Lynch JP (2004). Cdxl or Cdx2 expression activates E-cadherin-ediated cell-cell adhesion and compaction in human colo 205 cells. Am J Physiol Castrointest Liver Physiol.

[27] Werling RW, Yaziji H, Bacchi CE, Gown AM (2003). CDX2, a highly sensitive and specific marker of adenocarcinomas of intestinal origin: an immunohistochemical survey of 476 primary and metastatic carcinomas. Am J Surg Pathol.

[28] Zheng J, Sun X, Wang W, Lu S (2010). Hypoxia-inducible factor-1α modulates the down-regulation of the homeodomain protein CDX2 in colorectal cancer. Oncol Rep.

[29] Dang LH, Chen F, Ying C, Chun SY, Knock SA, Appelman HD, Dang DT (2006). CDX2 has tumorigenic potential in the human colon cancer cell lines Lovo and SW48. Oncogene.

[30] Zheng JB, Sun XJ, Li SS, Wang W, Ren HL, Tian Y, Lu SY, Du JK (2011). Effects of homeodomain protein CDX2 expression on the proliferation and migration of lovo colon cancer cells. Pathol Oncol Res.

[31] Gross I, Duluc I, Benameur T, Calon A, Martin E, Brabletz T, Kedinger M, Domon-Dell C, Freund JN (2008). The intestine-specific homeobox gene Cdx2 decreases mobility and antagonizes dissemination of colon cancer cells. Oncogene.

[32] Benahmed F, Gross I, Guenot D, Jehan F, Martin E, Domon-Dell C, Brabletz T, Kedinger M, Freund JN, Duluc I (2007). The microenvi-ronment controls CDX2 homeobox gene expression in colorectal cancer cells. Am J Pathol.

[33] Hanahan D, Weinberg RA (2000). The hallmarks of cancer. Cell.

[34] Derbal-Wolfrom L, Pencreach E, Saandi T, Aprahamian M, Martin E, Greferath R, Tufa E, Choquet P, Lehn JM, Nicolau C, Duluc I, Freund JN (2013). Increasing the oxygen load by treatment with myo-inositol trispyrophosphate reduces growth of colon cancer and modulates the intestine homeobox gene Cdx2. Oncogene.

[35] Jiang WG, Sanders AJ, Katoh M Tissue invasion and metastasis: Molecular, biological and clinical perspectives. Semin Cancer Biol.

[36] Murray GI (2001). Matrix metalloproteinasers: a multifunctional group of molecules. J Pathol.

[37] Mook OR, Frederiks WM, Van Noorden CJ (2004). The role of gelatinases in colorectal progression and metastasis. Biochim Biophys Acta.

[38] Li HC, Cao DC, Liu Y, Hou YF, Wu J, Lu JS, Di GH, Liu G, Li FM, Ou ZL (2004). Prognostic value of matrix metalloproteinases (MMP-2 and MMP-9) in patients with lymph nodenegative breast carcinoma. Breast Cancer Res Treat.

[39] Barbarosos A, Biacchi D, Bolognese A, Galati G, Izzo L, Risuleo G, Tartaglia E (2005). Molecular epidemiologic analysis of the levels of metalloproteinases and cyclooxygenase-2 in colorectal cancer. Supp Tumor.

[40] Baker EA, Leaper DJ (2003). The plasminogen activator and matrix metalloproteinase systems in colorectal cancer: relationship to tumor pathology. Eur J Cancer.

[41] Bodey B, Bodey B, Siegel SE, Kaiser HE (2000). Prognostic significance of matrix metalloproteinase expression in colorectal carcinoma. In vivo (Athens, Greece).

[42] Curran S, Dundas SR, Buxton J, Leeman MF, Ramsay R, Murray GI (2004). Murray matrix metalloproteinase/tissue inhibitors of matrix metalloproteinase phenotype identifies poor prognosis colorectal cancers. Clinic Can Res.

[43] Pesta M, Holubec L, Topolcan O, Cerna M, Rupert K, Holubec LS, Treska V, Kormunda S, Elgrova L, Finek J, Cerny R (2005). Quantitative estimation of matrix metalloproteinases 2 and 7 (MMP-2, MMP-7) and tissue inhibitors of matrix metal-loproteinases 1 and 2 (TIMP-1, TIMP-2) in colorectal carcinoma tissue samples. Anticancer Res.

